# Multipath Credibility Selection for Robust UWB Angle-of-Arrival Estimation in Narrow Underground Corridors

**DOI:** 10.3390/s26062002

**Published:** 2026-03-23

**Authors:** Jianjia Li, Baoguo Yu, Songzuo Cui, Menghuan Yang, Jun Zhao, Runjia Su, Runze Tian

**Affiliations:** The 54th Research Institute of China Electronics Technology Group Corporation, Shijiazhuang 050081, China; jianjia_li@126.com (J.L.);

**Keywords:** ultra-wideband (UWB), angle of arrival (AoA), multipath, narrow corridor, underground logistics tunnel, MUSIC, phase difference of arrival (PDOA)

## Abstract

Waveguide-like propagation in elongated underground environments—utility corridors, logistics tunnels—generates dense multipath that can cause the earliest or strongest resolvable channel impulse response (CIR) component to originate from a specular reflection rather than the direct line-of-sight (LOS) path. In the single-anchor CIR-tap-based implementations common to practical ultra-wideband (UWB) systems, baseline estimators such as phase-difference-of-arrival (PDOA) and MUSIC rely on selecting a single dominant CIR component, producing large angle-of-arrival (AoA) errors whenever the selected path is a reflection. We propose a multipath credibility selection (MCS) AoA estimator, MCS-AoA, that does not require explicit LOS/NLOS classification. The algorithm scores each resolvable CIR component with four credibility factors—amplitude significance, time-of-flight (TOF) consistency, inter-baseline phase–geometry agreement, and cross-baseline coherence—and fuses retained candidates into a credibility-weighted spatial covariance matrix for 2D MUSIC search. Field experiments on a custom five-channel coherent UWB platform compare MCS-AoA against six baselines—PDOA, MUSIC, MVDR/Capon, TLS-ESPRIT, PwMUSIC, and DNN-AoA. In an underground corridor (5–40 m), MCS-AoA achieves an azimuth/elevation MAE of 1.00°/1.46°, outperforming all baselines (PDOA: 2.26°/2.49°; MUSIC: 1.76°/2.40°; next-best PwMUSIC: 1.44°/2.17°); in a logistics tunnel (5–80 m), it achieves a 1.19° overall azimuth MAE. Simulations corroborate these gains, with a 0.71° azimuth RMSE at 80 m (69.3% reduction over PDOA) and 86.6% of estimates falling within 1°.

## 1. Introduction

Underground corridors and logistics tunnels are a natural fit for single-anchor ultra-wideband (UWB) localization: infrastructure inspection, safety monitoring, and emergency response all benefit from deploying only one fixed node. The chief difficulty is multipath. Waveguide-like propagation in these elongated spaces produces dense specular and diffuse reflections. The strongest or earliest resolvable channel impulse response (CIR) component can therefore be a reflected path rather than the direct line-of-sight (LOS). In the single-anchor CIR-tap-based implementations used here, the compared baselines rely on selecting a single dominant CIR component that is presumed to correspond to the LOS, typically the earliest detectable peak or the strongest CIR tap. A single erroneous selection yields an angular bias on the order of degrees, and the error distribution develops heavy tails that worsen with increasing range.

This paper takes a different approach: rather than committing to one path, it treats every resolvable CIR component as a candidate observation and assigns each a data-driven weight. The resulting algorithm, multipath credibility selection AoA (MCS-AoA), scores candidates with a four-dimensional credibility metric, assembles a credibility-weighted spatial covariance matrix, and resolves azimuth and elevation via a 2D MUSIC search. Simulation and field measurements in a utility corridor (5–40 m) and a logistics tunnel (5–80 m) show that MCS-AoA reduces the MAE by 41–56% relative to PDOA and 36–43% relative to MUSIC across both sites.

## 2. Related Work

The propagation environment targeted by this work differs from typical indoor channels in two measurable respects: excess delay and relative multipath amplitude. Zhou et al. [1] showed through physics-based deterministic UWB channel models that waveguide-like tunnel propagation generates multipath structures with low excess delay and high relative amplitude—characteristics that violate the assumptions underlying most indoor-oriented algorithms. Experimental confirmation comes from Nkakanou et al. [2], who measured UWB channel parameters in underground mine galleries and characterized path loss, coherence bandwidth, and delay spread under both LOS and NLOS conditions. Hrovat et al. [3] surveyed the broader landscape of tunnel propagation modeling, spanning numerical, waveguide/modal, ray-tracing, and empirical approaches, while Bashir [4] examined the influence of antenna position and polarization on UWB signals in mine tunnels.

Against this propagation backdrop, AoA estimation has traditionally relied on two families of algorithms. Phase-based methods such as PDOA require minimal computation but break down when multipath perturbs the inter-channel phase by more than a fraction of a wavelength. Subspace methods achieve finer angular resolution: the MUSIC algorithm proposed by Schmidt [5] exploits signal–noise subspace orthogonality for super-resolution DOA estimation, ESPRIT [6] avoids explicit spectral search through a rotation-invariance formulation, and the MVDR beamformer [7] provides a constrained spatial–spectrum estimate. Joint azimuth–elevation estimation has been demonstrated with L-shaped arrays by Porozantzidou and Chryssomallis [8]. Yet, subspace methods remain vulnerable when multipath contaminates the sample covariance matrix, as is common in confined underground spaces. Deng et al. [9] addressed the computational cost of 2D MUSIC by decomposing the joint peak search into two sequential 1D searches, but the underlying sensitivity to covariance corruption remains. An alternative paradigm was explored by Ledergerber and D’Andrea [10], who estimated AoA from the angle-dependent antenna transfer function of a single UWB element, augmenting TOF systems with angular information without a multi-element array.

How to handle multipath is the central open question. Existing strategies span four categories: (i) heuristic LOS detection via TOF or amplitude thresholds; (ii) model-based multipath parameter estimation; (iii) multipath-assisted localization that treats reflections as useful information; and (iv) robust covariance construction with weighted fusion. The SALMA framework [11] exemplifies category (iii), mapping specular multipath components to virtual anchors for single-anchor positioning even under obstructed LOS. Wielandt and De Strycker [12] pursued a related idea, using ray tracing to generate multipath-assisted AoA fingerprints. Nguyen et al. [13] combined geometry-based features with Gaussian-process regression to model resolvable specular paths from UWB CIR data, while Hu et al. [14] fused NLOS delay and angular measurements through ray tracing and iterative reweighted least squares for underground parking scenarios.

A parallel research thread focuses on distinguishing LOS from NLOS propagation before estimation. Marano et al. [15] pioneered learning-based NLOS classifiers from large-scale UWB campaigns; Zeng et al. [16] used hand-crafted CIR features for the same purpose. Deep learning variants followed: Stahlke et al. [17] applied convolutional networks directly to raw CIR waveforms, and Jiang et al. [18] trained classifiers on CIR features in complex indoor settings. Guvenc and Chong [19] provide a comprehensive survey of TOA-based NLOS mitigation. These classifiers improve ranging but do not directly address AoA bias introduced when a non-LOS component dominates the covariance.

Foundational analyses of wideband localization accuracy by Shen and Win [20], Gezici et al. [21], and Dardari et al. [22] highlight multipath interference and NLOS bias as dominant error sources. Physical-layer principles were laid out by Win and Scholtz [23]; channel modeling aspects were surveyed by Molisch [24]. Practical PDOA-based AoA estimation with commercial Decawave DW1000 hardware was demonstrated by Dotlic et al. [25], and Smaoui et al. [26] explored concurrent AoA/ranging architectures. Compact UWB arrays for AoA-aided relative localization were reported by Mathew et al. [27], and cooperative strategies were studied by Wymeersch et al. [28].

In elongated underground spaces, the bottleneck is not multipath per se but the inability to decide which path to trust. The present work addresses this gap by assigning every candidate a credibility score derived from array geometry and TOF constraints; the weighted covariance construction then emphasizes reliable paths automatically, without a LOS detector or offline training data.

## 3. Materials and Methods

### 3.1. Signal Model and Virtual Array Interpretation

In a confined underground environment, specular reflections frequently produce resolvable multipath components that maintain stable phase relationships across the antenna array. These components can be interpreted as observations originating from a set of multipath-induced virtual array (MIVA) elements. For a physical array whose element positions are ${\left\{p_{m}\right\}}_{m=1}^{M}$, each specular multipath component can be modeled as an equivalent virtual displacement $\Delta r_{\ell }$ along the direction of incidence, where

(1) $$\Delta r_{\ell }=c\;\tau_{\ell },$$

and *c* is the speed of light and $\tau_{\ell }$ is the corresponding excess delay.

For a candidate path *ℓ* with incident direction parameterized by azimuth/elevation (ϕ,θ), the array steering vector is

(2) $$a\left(\phi ,\theta \right)={\left[\exp\left(jk_{0}\;p_{1}^{T}u\right),\ldots ,\exp\left(jk_{0}\;p_{M}^{T}u\right)\right]}^{T},$$

where $k_{0}=2\pi /\lambda$ is the free-space wavenumber and $u={\left[\sin\phi \cos\theta ,\cos\phi \cos\theta ,-\sin\theta \right]}^{T}$ is the unit direction vector. When multiple resolvable multipath components are present, the received signal can be interpreted as an extended (virtual) array response obtained by stacking the steering vectors of the individual virtual elements:

(3) $$a_{\mathrm{ext}}\left(\phi ,\theta \right)={\left[a_{1}^{T}\left(\phi ,\theta \right),a_{2}^{T}\left(\phi ,\theta \right),\ldots ,a_{K}^{T}\left(\phi ,\theta \right)\right]}^{T},$$

where *K* is the number of resolvable candidate paths and $a_{k}\left(\phi ,\theta \right)$ denotes the steering vector associated with the *k*-th virtual element.

For a linear baseline, the phase at the *ℓ*-th virtual element on the *m*-th antenna reduces to the classical form $\varphi_{\ell }^{\left(m\right)}=\left(2\pi /\lambda \right)\;d_{m}\sin\theta_{\ell }$, underscoring that resolvable multipath components can serve as additional “virtual sensors” that effectively extend the array aperture.

#### 3.1.1. Virtual-Array Resolvability

For a virtual element to contribute non-degenerate angular information, a necessary condition is that its steering vector remains linearly independent of the physical array steering vector over the angular region of interest, i.e.,

(4) $$\mathrm{rank}\left([a\left(\phi ,\theta \right),\;a_{\mathrm{virt}}\left(\phi ,\theta \right)]\right)=2.$$

For uniform linear arrays with element spacing *d* and *M* elements, a heuristic sufficient condition can be derived as follows. The additional phase shift introduced by the *ℓ*-th virtual element relative to the physical array reference is $\Delta \Phi_{\ell }=\left(2\pi /\lambda \right)\;\Delta r_{\ell }\sin\theta$, where θ is the elevation angle of incidence. Steering-vector degeneracy occurs when this phase shift equals an integer multiple of 2π, i.e., $\Delta r_{\ell }\sin\theta =n\lambda$ for some n∈Z. To avoid degeneracy, the residual phase must exceed the array’s angular resolution limit. For an *M*-element ULA with total aperture (M−1)d, the Rayleigh-like angular resolution is $\Delta \theta_{\min}\approx \lambda /\left(2(M-1)d\right)$, which corresponds to a minimum distinguishable phase separation of $\delta \Phi_{\min}=\left(2\pi /\lambda \right)\;\left(M-1\right)d\;\Delta \theta_{\min}=\pi$, or equivalently a path-length margin of λ/(2M) when normalized by the number of independent baselines. This yields the condition

(5) $$\left|\Delta r_{\ell }\;\sin\theta -n\lambda \right|>\frac{\lambda }{2M},\;\forall n\in Z,$$

which ensures that the virtual element’s contribution remains distinguishable from integer-wavelength phase aliases within the array’s resolution capability.

In practice, however, not all resolvable components in underground environments satisfy the geometric and phase relations required by the MIVA interpretation. Such components act as “false virtual elements” that corrupt the estimated covariance matrix. This contamination can be captured by a low-rank bias term,

(6) $$\hat{R}=R_{\mathrm{true}}+\sigma_{\mathrm{err}}^{2}\;a_{\mathrm{err}}a_{\mathrm{err}}^{H},$$

which violates the signal–noise subspace orthogonality upon which subspace estimators rely. MCS-AoA counteracts this effect by assigning low credibility scores to candidates that violate phase–geometry and coherence constraints, thereby suppressing their contribution to the weighted covariance matrix.

#### 3.1.2. Validity Under Diffuse Multipath Conditions

The virtual array model assumes specular reflection, i.e., each multipath component arrives from a well-defined direction with a coherent wavefront. In practice, underground environments also exhibit diffuse scattering from rough surfaces, cabling, and ventilation infrastructure. A diffuse component lacks a stable arrival angle and therefore violates the plane-wave assumption underlying the steering vector a(ϕ,θ).

The MCS credibility framework handles such components without requiring an explicit specular/diffuse classifier. Diffuse arrivals typically exhibit (i) low inter-baseline phase consistency, because the wavefront curvature varies across the array aperture, producing a low phase–geometry score $S_{\mathrm{geo}}^{\left(\ell \right)}$; (ii) low cross-baseline coherence $S_{\mathrm{coh}}^{\left(\ell \right)}$, because the scattered field decorrelates across spatially separated baselines; and (iii) temporally spread energy that reduces the peak amplitude relative to the noise floor, lowering $S_{\mathrm{amp}}^{\left(\ell \right)}$. Consequently, diffuse components receive low composite credibility $C_{\ell }$ and are effectively suppressed by the adaptive threshold $\eta^{★}$. The method therefore does not rely on a strictly specular propagation environment; rather, it degrades gracefully as the diffuse-to-specular ratio increases, retaining only those components whose physical consistency survives the four-dimensional credibility test.

### 3.2. Resolvable Condition and Soft-Decision Fusion

To retain only resolvable and physically consistent candidates, we adopt a soft-decision fusion strategy in lieu of hard LOS selection. In conventional estimators, the total AoA error decomposes into a path-selection term and an estimation term,

(7) $$\varepsilon_{\mathrm{trad}}=\varepsilon_{\mathrm{sel}}+\varepsilon_{\mathrm{est}}\left(\phi ,\theta \right),$$

where $\varepsilon_{\mathrm{sel}}$ can become a catastrophic outlier once an incorrect path is chosen. MCS-AoA instead constructs a credibility-weighted covariance matrix from multiple candidate snapshots and estimates AoA via a MUSIC spectrum search:

(8) $$\left(\hat{\phi },\hat{\theta }\right)=\arg\max_{\phi ,\theta }P_{\mathrm{MUSIC}}\left(\phi ,\theta \right),$$

where $P_{\mathrm{MUSIC}}\left(\phi ,\theta \right)$ is evaluated on the credibility-weighted covariance matrix formed from the retained candidate set S (detailed in the following subsections).

From an information-fusion perspective, the credibility weighting acts as a soft decision that down-weights unreliable candidates while emphasizing those exhibiting physical consistency.

### 3.3. Array Observation and Multipath Candidates

In confined underground spaces, the received CIR typically comprises multiple reflections of comparable energy. Let the CIR matrix be $R\in C^{N\times M}$, where *N* denotes the number of delay taps and *M* the number of array elements. At each delay index *k*, the corresponding array snapshot is $x\left(k\right)=R{\left(k,:\right)}^{T}\in C^{M}$.

Rather than committing to a single presumed LOS tap, we first extract a compact set of candidate paths through peak detection on a reference channel. Let $r_{1}=R\left(:,1\right)$ denote the magnitude sequence of the reference-channel CIR. Peaks are identified on $|r_{1}|$ using a relative amplitude threshold of $0.15\;\max|r_{1}|$, and at most L≤5 candidates are retained. If no peak exceeds the threshold, the global-maximum tap is used as the sole candidate.

### 3.4. Multipath Credibility Selection (MCS)

We adopt a Bayesian-inspired heuristic to assess the credibility of each candidate path. For each candidate ℓ∈{1,…,L} at delay index $k_{\ell }$, let $u_{\ell }\in \left\{0,1\right\}$ indicate whether it is a “usable” (physically consistent) virtual element and let $\rho_{\ell }=\left[\varepsilon_{\ell }^{\left(0\right)},\tau_{\ell },S_{\mathrm{amp}}^{\left(\ell \right)},S_{\mathrm{coh}}^{\left(\ell \right)}\right]$ collect the observed features—phase residual, excess delay, amplitude score, and coherence score. Under a naive-Bayes-style conditional independence assumption, the posterior would factorize as

(9) $$\begin{array}{cc} P(u_{\ell }=1|\rho_{\ell }) & \propto P\left(u_{\ell }=1\right)\;P\left(\varepsilon_{\ell }^{\left(0\right)}|u_{\ell }=1\right)\;P\left(\tau_{\ell }|u_{\ell }=1\right) \end{array}$$

(10) $$\begin{array}{cc} \; & \;\cdot P\left(S_{\mathrm{amp}}^{\left(\ell \right)}|u_{\ell }=1\right)\;P\left(S_{\mathrm{coh}}^{\left(\ell \right)}|u_{\ell }=1\right). \end{array}$$

This factored form motivates a multiplicative heuristic score that combines four physically grounded terms:

(11) $$C_{\ell }=S_{\mathrm{amp}}^{\left(\ell \right)}\;S_{\mathrm{tof}}^{\left(\ell \right)}\;S_{\mathrm{geo}}^{\left(\ell \right)}\;S_{\mathrm{coh}}^{\left(\ell \right)}.$$

Taking the logarithm and substituting the exponential forms of $S_{\mathrm{tof}}^{\left(\ell \right)}$ and $S_{\mathrm{geo}}^{\left(\ell \right)}$ yields

(12) $$\log C_{\ell }=\log\left(S_{\mathrm{amp}}^{\left(\ell \right)}+\epsilon \right)+\log\left(S_{\mathrm{coh}}^{\left(\ell \right)}+\epsilon \right)-\frac{\varepsilon_{\ell }^{\left(0\right)}}{\pi }-\alpha \;\left(k_{\ell }-k_{1}\right),$$

where $\epsilon =10^{-10}$ prevents numerical underflow when a factor vanishes. The additive log-domain decomposition shows that each credibility dimension contributes an independent surrogate log-likelihood term. Arithmetic averaging or maximum selection would break this factored structure and discard complementary information; the ablation study in Section 5.1 confirms that the multiplicative rule outperforms both alternatives by 25–26%.

Each factor captures a distinct physical consistency dimension. We extract the array snapshot $x_{\ell }=x\left(k_{\ell }\right)$ and compute:

Amplitude significance. The relative strength of candidate *ℓ* with respect to the strongest detected component:

(13) $$S_{\mathrm{amp}}^{\left(\ell \right)}=\frac{\left|R(k_{\ell },1)\right|}{\max_{k}\left|R\left(k,1\right)\right|}.$$

TOF proximity. An earliest-arrival prior penalizing late arrivals relative to the first detected peak at delay index $k_{1}$:

(14) $$S_{\mathrm{tof}}^{\left(\ell \right)}=\exp\left(-\alpha \;(k_{\ell }-k_{1})\right),$$

with α=0.1.

**Figure 1:**
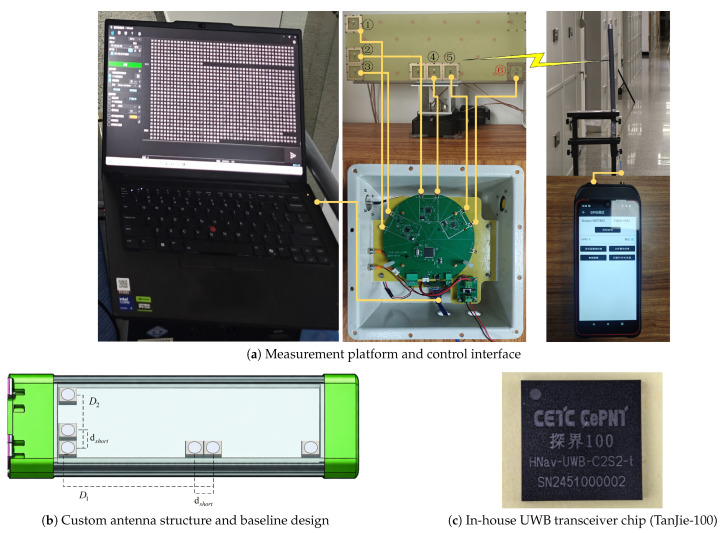
UWB AoA measurement platform. (**a**) Base station with control laptop, tag, and mobile interface; the graphical user interface is displayed in Chinese, which is the native language of the operating software. (**b**) L-shaped antenna array with baseline dimensions. (**c**) Close-up of the custom dual-channel UWB transceiver chip (CETC CePNT TanJie-100); three such chips provide the (1Tx+5Rx) configuration. The circled numbers ①–⑥ label the six antenna elements; the red numeral denotes the transmit element, which is used for ranging only and does not participate in AoA estimation.

Phase–geometry consistency. A residual measuring how well inter-baseline phase differences obey the integer-multiple relationships imposed by array geometry. For a uniform sub-baseline with equally spaced elements, the phase difference to the *b*-th element should satisfy $\Delta \varphi_{1b}^{\left(\ell \right)}\approx b\;\Delta \varphi_{12}^{\left(\ell \right)}$. The residual is

(15) $$S_{\mathrm{geo}}^{\left(\ell \right)}=\exp\;\left(-\frac{J_{\ell }}{\pi }\right),\;J_{\ell }=\frac{\sum_{b}c_{\ell ,b}\;\left|\mathrm{wrap}\left(\Delta \varphi_{1b}^{\left(\ell \right)}-b\;\Delta \varphi_{12}^{\left(\ell \right)}\right)\right|}{\sum_{b}\left(c_{\ell ,b}+\epsilon \right)},$$

where $\Delta \varphi_{1b}^{\left(\ell \right)}=∠\left(x_{b}^{\left(\ell \right)}\;{x_{1}^{\left(\ell \right)}}^{*}\right)$ is the wrapped phase difference between the reference antenna and the *b*-th element for candidate *ℓ*, and $c_{\ell ,b}\in \left[0,\;1\right]$ is a per-baseline reliability weight. In the general formulation, $c_{\ell ,b}$ can be set to the normalized cross-correlation magnitude of adjacent antenna pairs, $c_{\ell ,b}=\left|x_{b}^{\left(\ell \right)}\;{x_{b-1}^{\left(\ell \right)}}^{*}\right|/\left(|x_{b}^{\left(\ell \right)}\left|\;\right|x_{b-1}^{\left(\ell \right)}|+\epsilon \right)$; in the present implementation, uniform weighting $c_{\ell ,b}=1$ is used. For the L-shaped array (Figure 1b), the integer-multiple check is applied independently to each arm. Let the horizontal sub-array consist of three equally spaced receive elements $\left\{H_{1},H_{2},H_{3}\right\}$ and the vertical sub-array of $\left\{V_{1},V_{2},V_{3}\right\}$, with $H_{1}$ and $V_{1}$ sharing the corner element. The per-arm residuals are $e_{H}=\left|\mathrm{wrap}\left(\Delta \varphi_{H_{1}H_{3}}^{\left(\ell \right)}-2\Delta \varphi_{H_{1}H_{2}}^{\left(\ell \right)}\right)\right|$ and $e_{V}=\left|\mathrm{wrap}\left(\Delta \varphi_{V_{1}V_{3}}^{\left(\ell \right)}-2\Delta \varphi_{V_{1}V_{2}}^{\left(\ell \right)}\right)\right|$, giving $J_{\ell }=e_{H}+e_{V}$. We define $\varepsilon_{\ell }^{\left(0\right)}=J_{\ell }$ for notational convenience.

Cross-baseline coherence. The mean resultant length of the phase differences across all baselines, rewarding candidates with self-consistent spatial signatures:

(16) $$S_{\mathrm{coh}}^{\left(\ell \right)}=\left|\frac{1}{N_{b}}\sum_{b=1}^{N_{b}}\exp\left(j(\varphi_{\ell ,b}-{\bar{\varphi }}_{\ell })\right)\right|,\;{\bar{\varphi }}_{\ell }=\frac{1}{N_{b}}\sum_{b=1}^{N_{b}}\varphi_{\ell ,b}.$$

For the L-shaped array, $S_{\mathrm{coh}}^{\left(\ell \right)}$ is computed per-arm as the normalized magnitude of adjacent-pair cross-correlations, $\gamma_{\mathrm{arm}}=|\sum_{b}x_{b+1}^{\left(\ell \right)}\;{x_{b}^{\left(\ell \right)}}^{*}|/\left(\sum_{b}|x_{b+1}^{\left(\ell \right)}\left|\;\right|x_{b}^{\left(\ell \right)}|+\epsilon \right)$, and averaged over the horizontal and vertical arms: $S_{\mathrm{coh}}^{\left(\ell \right)}=\left(\gamma_{H}+\gamma_{V}\right)/2$. When phase progressions across consecutive elements are consistent, the cross-correlations add coherently and $S_{\mathrm{coh}}^{\left(\ell \right)}\to 1$; when phases are incoherent, cancellation drives $S_{\mathrm{coh}}^{\left(\ell \right)}\to 0$.

The composite credibility $C_{\ell }$ (equivalently, $\gamma_{\ell }=C_{\ell }$) is normalized to produce the fusion weight $w_{\ell }$. Table 1 summarizes the four credibility factors.

An adaptive credibility threshold η is further applied to suppress low-credibility candidates. Let $p_{\ell }=C_{\ell }/\left(\sum_{i=1}^{L}C_{i}+\epsilon \right)$ and $S\left(\eta \right)=\left\{\ell :\;C_{\ell }\geq \eta \right\}$. We define

(17) $$P_{\mathrm{miss}}\left(\eta \right)=\sum_{\ell \notin S(\eta )}p_{\ell },\;P_{\mathrm{fa}}\left(\eta \right)=\frac{1}{\left|S(\eta )\right|}\sum_{\ell \in S(\eta )}\left(1-p_{\ell }\right),$$

and select

(18) $$\eta^{★}=\arg\min_{\eta }\left(P_{\mathrm{miss}}\left(\eta \right)+\lambda_{\mathrm{risk}}\;P_{\mathrm{fa}}\left(\eta \right)\right),$$

where $\lambda_{\mathrm{risk}}$ governs the miss-versus-false-alarm trade-off. In practice, we perform a discrete search over the candidate credibility values and set $\lambda_{\mathrm{risk}}=0.5$, with the constraint that at least one candidate is always retained.

The normalized credibility weight is

(19) $$w_{\ell }=\frac{C_{\ell }}{\sum_{i\in S(\eta^{★})}C_{i}+\epsilon },\;l\in S\left(\eta^{★}\right).$$

### 3.5. Credibility-Weighted Covariance and 2D MUSIC Search

The credibility-weighted spatial covariance matrix is constructed from the retained candidate snapshots as

(20) $$R_{w}=\sum_{\ell =1}^{L}w_{\ell }\;x_{\ell }x_{\ell }^{H}.$$

An eigendecomposition $R_{w}=U\Sigma U^{H}$ yields the noise subspace $E_{n}$, spanned by the eigenvectors corresponding to the smallest eigenvalues. A single-source model (d=1) is assumed when partitioning the eigenspace, i.e., $E_{n}$ comprises the M−1 eigenvectors with the smallest eigenvalues. This assumption is justified by two observations: (i) the credibility thresholding retains only candidates whose phase–geometry and coherence scores are mutually consistent, so the surviving snapshots approximate observations of the same dominant arrival direction; and (ii) even when a secondary direction contributes residual energy, the credibility weighting concentrates most of $R_{w}$’s energy along the primary eigenvector, ensuring that the MUSIC null-steering remains well-directed. The 2D MUSIC pseudo-spectrum is then evaluated as

(21) $$P_{\mathrm{MUSIC}}\left(\phi ,\theta \right)=\frac{1}{a^{H}\left(\phi ,\theta \right)\;E_{n}E_{n}^{H}\;a\left(\phi ,\theta \right)},$$

and the AoA estimate is obtained via grid search:

(22) $$\left(\hat{\phi },\hat{\theta }\right)=\arg\max_{\phi ,\theta }P_{\mathrm{MUSIC}}\left(\phi ,\theta \right).$$

At longer ranges where the elevation estimation becomes ill-conditioned, the search can be reduced to a 1D azimuth scan by fixing θ or applying a 2D-to-1D dimensionality reduction.

### 3.6. Algorithm Flow

Table 2 summarizes the complete MCS-AoA processing pipeline.

### 3.7. Implementation Details and Parameter Settings

For reproducibility, we summarize the key parameter settings of our implementation. Candidate extraction relies on peak detection applied to the reference-channel magnitude $|r_{1}|$ with a relative threshold of $0.15\;\max|r_{1}|$; at most, L=5 candidates are retained, with the global-maximum tap serving as a fallback when no peak exceeds the threshold. An earliest-arrival prior is imposed via

(23) $$S_{\mathrm{tof}}^{\left(\ell \right)}=\exp\left(-\alpha \;(k_{\ell }-k_{1})\right),$$

where $k_{1}$ is the delay index of the earliest detected peak and α=0.1. The phase wrapping operator wrap(·) maps its argument to [−π,π). In the 2D MUSIC step, all available receive channels are used and a single-source model is assumed. Crucially, the same parameter configuration (Table 3) is applied across all simulation and experimental scenarios without per-environment tuning, ensuring that the reported results reflect generalization capability rather than scenario-specific overfitting.

### 3.8. Computational Complexity

Let *M* denote the number of receive channels, *L* the number of retained candidates, and *G* the total number of grid points in the 2D scan. Constructing the credibility-weighted covariance $R_{w}$ requires $O(LM^{2})$ operations, the eigendecomposition costs $O(M^{3})$, and evaluating the MUSIC pseudo-spectrum over the scan grid costs $O(GM^{2})$. With the grid specified in Table 3, G=121×71=8591, so the spectral evaluation dominates the overall computational burden.

Measured execution time. On a desktop PC (Intel Core i7-12700, 2.1 GHz, 32 GB RAM) running unoptimized MATLAB (R2023a), a single MCS-AoA estimation cycle—including peak detection, four-dimensional credibility evaluation, covariance construction, eigendecomposition, and full 2D grid search—completes in approximately 12 ms per CIR snapshot. This is well within the 100 ms budget imposed by the 10 Hz AoA update rate of the measurement platform described in Section 4.1, confirming real-time feasibility even without code optimization.

Acceleration strategies. For deployment on resource-constrained embedded platforms, the computational cost can be reduced through several strategies: (i) a *coarse-to-fine* grid search that first evaluates a sparse grid (e.g., 5° steps) and then refines around the coarse peak with 0.5° resolution, reducing *G* by roughly an order of magnitude; (ii) restricting the candidate count to L≤3 when prior position information is available; and (iii) exploiting the Hermitian structure of $R_{w}$ to halve the number of complex multiplications in the spectrum evaluation. A combination of these techniques is expected to bring the per-cycle latency below 2 ms on an ARM Cortex-A class processor, enabling integration into real-time UWB localization stacks.

## 4. Experimental Setup

### 4.1. UWB AoA Platform

The UWB AoA measurement system is a fully custom-developed radio platform rather than a development board or commercial module; no commercial UWB transceiver IC (e.g., Qorvo DW1000/DW3000) is used. The base station is built around three in-house-designed dual-channel UWB transceiver chips (CETC CePNT *TanJie-100*, see Figure 1c), which together provide one transmit channel and five coherent receive channels in a (1Tx+5Rx) configuration. Each transceiver chip integrates a zero-IF (direct-conversion) RF front-end and a 1.5-bit analog-to-digital converter (ADC). The receive chain of each channel comprises a low-noise amplifier (LNA, 15 dB gain, 1.7 dB noise figure), an in-phase/quadrature (I/Q) down-conversion mixer, and a variable-gain amplifier (VGA, 19.5 dB gain, 25 dB adjustable range), yielding a system noise figure of approximately 2.3 dB and a receiver sensitivity of approximately −101 dBm. The transmit chain consists of a pulse generator (pulse width ≈2 ns, jitter <10 ps RMS), an adjustable-gain driver (31.5 dB dynamic range), an up-conversion mixer, and a power amplifier (15 dB gain, $P_{1\mathrm{dB}}$ = 13 dBm), with the EIRP limited to −14.3 dBm per the FCC 500 MHz emission mask.

All five receive channels share a common 38.4 MHz crystal oscillator, from which the local-oscillator and sampling clocks are derived, ensuring inter-channel clock synchronization with <50 ps jitter. The six-path ADC sampling subsystem operates at an equivalent-time sampling rate of 1996.8 MHz (approximately 4× the signal bandwidth). Although the raw ADC quantization is 1.5-bit, coherent accumulation of approximately 91,800 pulses per measurement epoch (mean PRF = 918 kHz, AoA update rate = 10 Hz) substantially increases the effective CIR amplitude resolution, yielding the complex I/Q output described in Section 4.1.2. A baseband processing module performs pulse detection, CIR accumulation, TOA/PDOA estimation, and data logging. The system operates at a center frequency of 3.9936 GHz (IEEE 802.15.4z HRP-UWB channel 5) with 499.2 MHz bandwidth, a mean PRF of 918 kHz, and a 10 Hz AoA update rate. The waveform is compatible with the IEEE 802.15.4z HRP-UWB standard.

#### 4.1.1. Inter-Channel Phase Calibration

Because AoA accuracy hinges on inter-channel phase coherence, all five Rx channels are clocked from the same 38.4 MHz crystal oscillator, thereby eliminating inter-channel clock drift. Residual per-channel phase offsets introduced by RF trace-length mismatches and component tolerances are removed through a factory calibration procedure: a reference transmitter is positioned at broadside (ϕ=0°, θ=0°) at a range of 5 m (satisfying the far-field condition), and the measured inter-channel phase differences are stored as static offset corrections that are subtracted from all subsequent measurements. This broadside calibration principle is analogous to the procedure described for commercial DW3000-based systems [29] but is implemented entirely on the custom transceiver hardware described above.

#### 4.1.2. CIR Data Format

Each measurement epoch yields a CIR matrix $R\in C^{N\times M}$, where *N* is the number of delay taps and M=5 is the number of receive channels. The CIR is stored in complex in-phase/quadrature (I/Q) format with an effective temporal resolution of approximately 1 ns. Each channel records up to 1016 taps, spanning a delay window of approximately 1 μs. Accompanying metadata includes a timestamp, the estimated first-path index, and the received signal strength indicator (RSSI) for each channel.

#### 4.1.3. Antenna Array Design

The L-shaped antenna array comprises six elements arranged in an L configuration—one transmit and five receive antennas. The detailed array parameters are listed in Table 4.

The array geometry follows a multi-baseline design strategy. Azimuth ϕ and elevation θ are jointly estimated from the five receive channels via a 2D spectrum search, and the multi-baseline configuration provides redundant phase-difference measurements that help distinguish true spatial signatures from multipath artifacts.

The AoA receiver shares the same core modules across all experiments—namely the UWB RF front-end, antenna array, clock/synchronization subsystem, and data-logging interface. To accommodate the varying deployment constraints encountered in different underground spaces (e.g., mounting height, mobility, protection class, and alignment accessibility), different mechanical enclosures were used while the signal chain and processing pipeline remained identical.

### 4.2. Underground Corridor Test

The corridor experiment spans distances from 5 m to 40 m at eight measurement points. The azimuth scan range is ϕ∈[−30°,+30°]: seven azimuth settings are tested per distance (−30° to +30° in 10° increments), and 30 CIR snapshots are acquired at each distance–azimuth combination, yielding 210 samples per distance and 1680 samples in total.

#### Angular Ground Truth

The angular ground truth was established with a high-precision total station (angular accuracy $\pm 1^{\prime \prime }$; distance accuracy ±(1mm+1.5ppm)). Transmitter and receiver positions were surveyed at every measurement point, and the ground-truth azimuth ϕ and elevation θ were derived from the surveyed coordinates. For elevation, the geometric relation $\theta =\arctan(h_{\mathrm{diff}}/r)$ was employed, where $h_{\mathrm{diff}}$ is the measured height difference between Tx and Rx and *r* is the horizontal separation. Given the total station’s measurement precision, the angular uncertainty of the derived ground-truth AoA is estimated at ±0.1° or better for ranges exceeding 5 m.

### 4.3. Underground Logistics Tunnel Test

**Figure 2:**
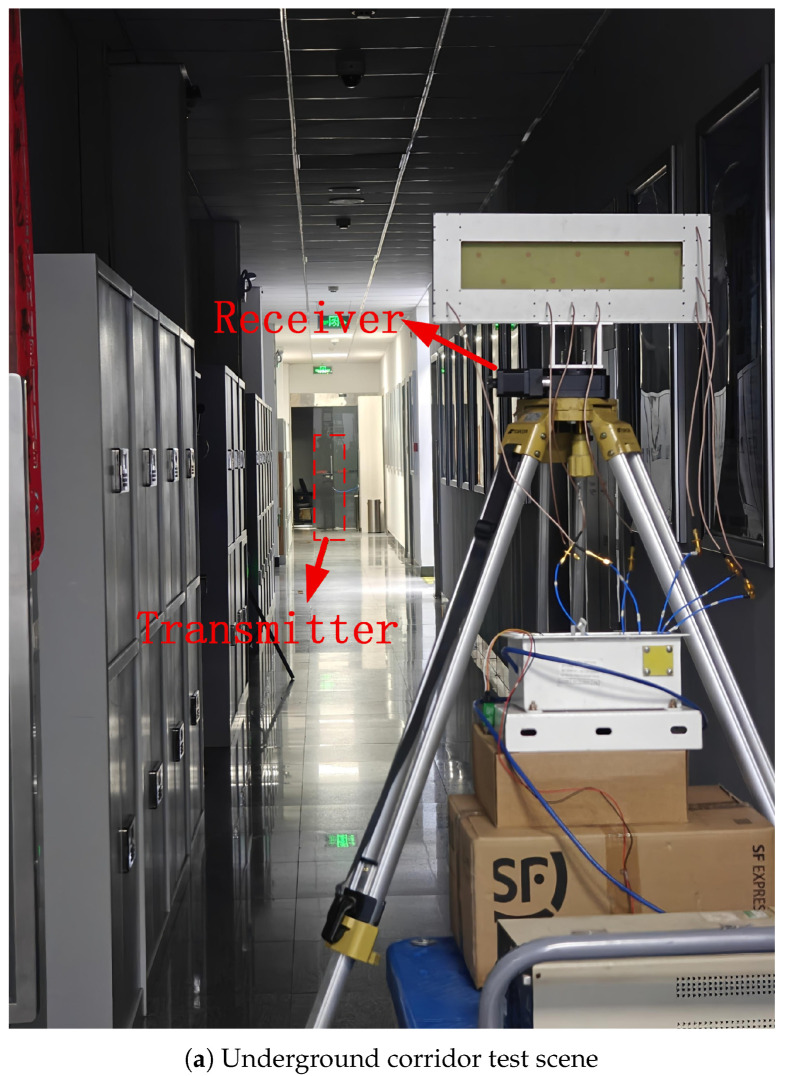

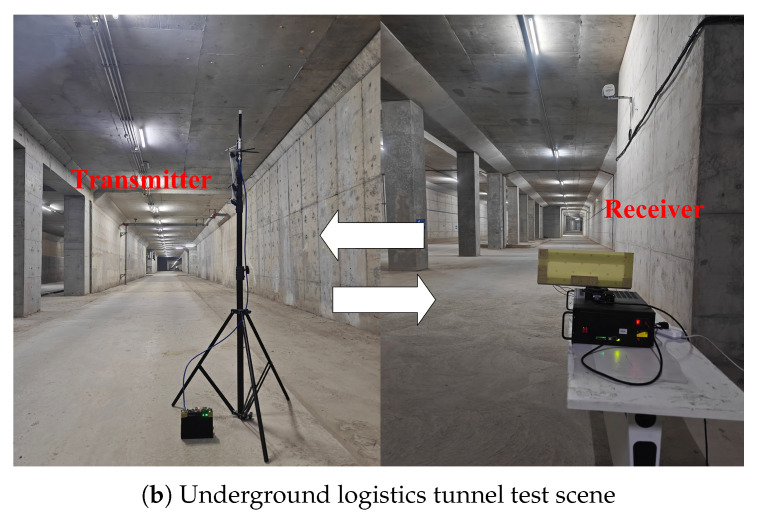
Field measurement environments. (**a**) Underground corridor deployment using the laboratory-grade enclosure shown in Figure 1. (**b**) Underground logistics tunnel deployment using a ruggedized enclosure specifically designed for harsh underground conditions. Both configurations share the same chip-based six-channel (1Tx+5Rx) RF front-end, antenna array, and signal processing pipeline; only the mechanical housing differs to accommodate the respective deployment requirements.

The underground logistics tunnel experiment extends the measurement range to 5–80 m. Both azimuth and elevation are reported for 5–40 m, while only azimuth is reported for 45–80 m owing to the diminishing elevation angular span at longer ranges. The data-collection protocol mirrors that of the corridor test: seven azimuth settings in 10° increments with 30 CIR snapshots per distance–azimuth combination (210 samples per distance). Figure 2 shows the two deployment environments.

## 5. Results

### 5.1. Simulation Results

Before turning to field data, we stress-test MCS-AoA under controlled multipath conditions using a parametric narrow-corridor channel model. The model generates 5–8 resolvable components with excess delays of 2–20 ns and amplitudes within 6 dB of the direct path—reproducing the low-delay, high-amplitude multipath characteristic of waveguide-like underground propagation, where wall reflections arrive close behind the LOS and can easily masquerade as the direct path. The post-correlator SNR is set to approximately 15 dB (pre-correlator ≈−10 dB given the UWB processing gain). Hardware non-idealities (ADC quantization, clock jitter, mutual coupling) are deliberately excluded; the field experiments in the following subsections capture these effects on real CIR data.

**Figure 3:**
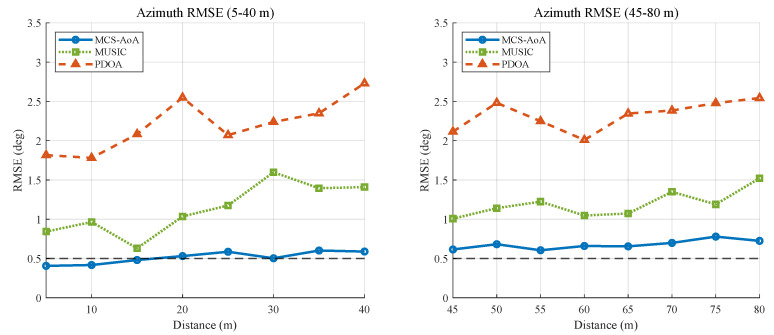
Simulation: azimuth RMSE versus distance (5–40 m and 45–80 m).

The most operationally relevant metric is distance-dependent accuracy, since underground localization must function over extended ranges. Figure 3 plots the azimuth RMSE from 5 m to 80 m (elevation is omitted beyond 40 m because the angular span falls below the achievable resolution). MCS-AoA remains below 1° RMSE across the full range, while the RMSE of MUSIC and PDOA increases steadily with distance. Table 5 further quantifies this comparison at representative distances.

**Figure 4:**
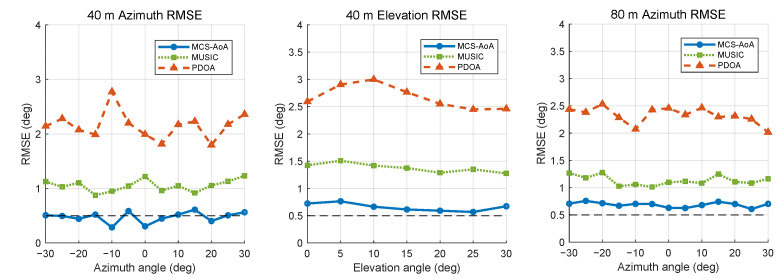
Simulation: RMSE versus incident angle (40 m azimuth/elevation and 80 m azimuth).

An angular sweep within ±30° (Figure 4) complements the distance analysis: at both 40 m and 80 m, MCS-AoA remains below 1° RMSE over the entire angular range, whereas the PDOA error grows with off-broadside angle.

**Figure 5:**
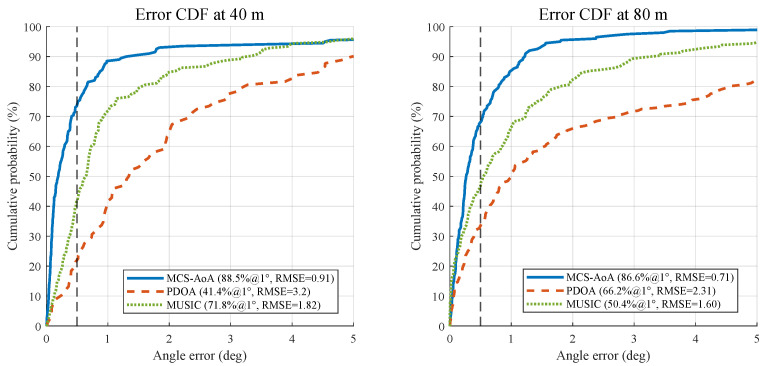
Simulation: AoA error CDFs at representative distances.

Figure 5 shows error CDFs at representative distances. At 40 m and 80 m, 88.5% and 86.6% of MCS-AoA estimates fall within 1°, respectively.

#### 5.1.1. Credibility Fusion Ablation Study

To validate the multiplicative credibility fusion rule, we compare three alternative strategies under identical simulation conditions (5–40 m, 30 Monte Carlo trials per point, median-per-point evaluation): (i) multiplicative (proposed), (ii) arithmetic mean (additive), and (iii) maximum selection (best-of-four). Table 6 reports the resulting MAE values. The multiplicative rule achieves the lowest azimuth MAE (1.10°), outperforming the additive rule (1.37°, +25%) and the maximum rule (1.39°, +26%), confirming that the product form retains complementary information from all four credibility dimensions.

#### 5.1.2. Hyperparameter Sensitivity Analysis

Table 7 reports the azimuth MAE obtained when each hyperparameter is varied individually while the remaining parameters are held at their default values (Table 3). The peak-detection threshold and the risk weight $\lambda_{\mathrm{risk}}$ change the MAE by less than 0.1°. The TOF decay factor α and the maximum candidate count *L* have a larger but bounded effect: varying α from 0.01 to 0.50 shifts the MAE from 1.86° to 0.71°, and varying *L* from 2 to 10 shifts it from 0.90° to 1.10°. Larger α values aggressively penalize late-arriving paths, which is beneficial in simulations where the LOS is always present but may prove overly restrictive under real-world NLOS conditions; α=0.1 thus represents a conservative operating point. Likewise, L≥5 ensures that sufficient candidate diversity is preserved; the marginal advantage of L=2 in simulation reflects idealized LOS availability and does not generalize to field conditions.

#### 5.1.3. Extended Baseline Comparison

To establish a broader benchmarking context, we compare MCS-AoA against five baselines under identical simulation conditions: PDOA, conventional MUSIC [5], the MVDR/Capon beamformer [7], TLS-ESPRIT [6], and power-weighted MUSIC (PwMUSIC), in which the credibility weights are replaced by squared-amplitude weights. Table 8 summarizes the resulting MAE values.

MCS-AoA achieves the best elevation accuracy (0.60°) among all methods. In azimuth, ESPRIT attains a lower MAE (0.60°) than MCS-AoA (1.10°) in this controlled setting because ESPRIT benefits from idealized LOS conditions in which the dominant eigenvalue corresponds to the true source direction. However, ESPRIT’s elevation accuracy is notably degraded (0.96°) owing to the limited vertical aperture, and its performance is expected to deteriorate in real-world scenarios where reliable LOS identification cannot be guaranteed. In azimuth, MVDR and PwMUSIC both perform worse than conventional MUSIC, as their sample-covariance-based and amplitude-only weighting strategies are more susceptible to multipath contamination of the covariance matrix (PwMUSIC does achieve competitive elevation accuracy, but its azimuth degradation is the more operationally significant limitation). Note that Table 5 reports RMSE at individual long-range distances (40 and 80 m), whereas Table 8 reports median-per-point MAE aggregated over the full 5–40 m span; the two metrics are not directly comparable. Sparse reconstruction and deep learning-based methods were excluded because they require either dense spatial sampling or representative training data, neither of which is available in our narrow underground deployment.

### 5.2. Underground Corridor (5–40 m)

**Figure 6:**
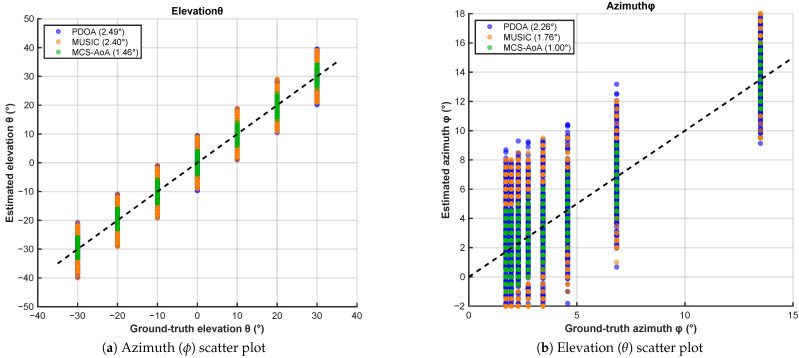
Underground corridor: AoA estimation scatter plots (5–40 m).

We first evaluate MCS-AoA in an underground corridor characterized by strong multipath and waveguide-like propagation. Figure 6a,b present the azimuth and elevation scatter plots of estimated versus ground-truth angles. MCS-AoA places the majority of estimates within a ±2° band around the diagonal reference line.

Table 9 summarizes the mean absolute error (MAE) across all corridor samples for seven methods evaluated on identical CIR data. MCS-AoA achieves the lowest joint azimuth–elevation MAE (1.00°/1.46°), followed by PwMUSIC (1.44°/2.17°). TLS-ESPRIT (1.76°/2.34°) and MVDR/Capon (1.79°/2.49°) perform comparably to conventional MUSIC (1.76°/2.40°); despite their theoretical resolution advantages, the dense multipath in this environment contaminates the sample covariance too severely for these methods to outperform MCS-AoA. The data-driven DNN-AoA baseline—trained via leave-one-distance-out cross-validation on a two-layer MLP with 64 hidden units—yields an azimuth MAE of 1.63° and an elevation MAE of 2.66°, ranking last among the seven methods in elevation, which indicates that the limited training corpus (∼1500 samples) is insufficient for robust generalization across the distance-dependent CIR variations in this confined environment.

**Figure 7:**
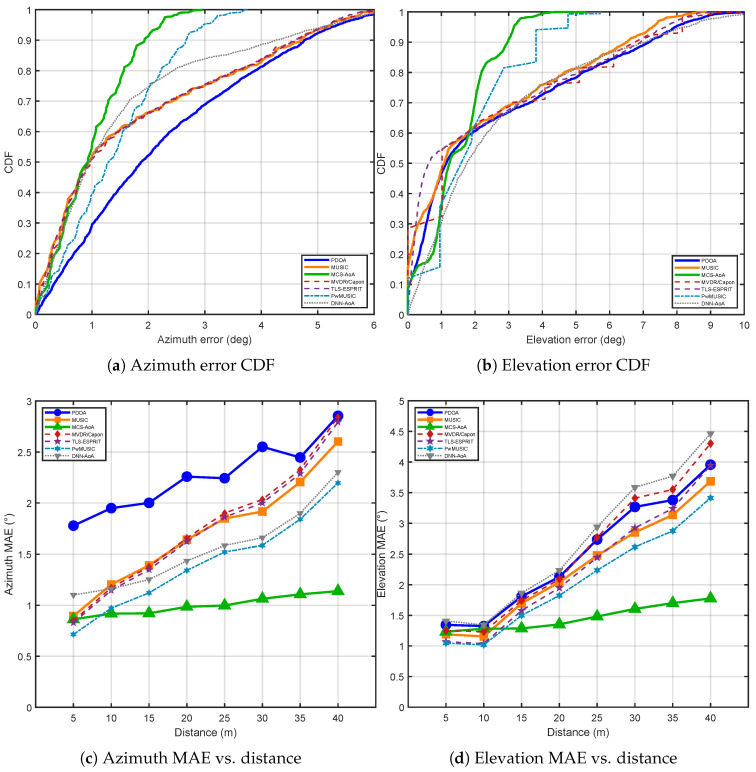
Underground corridor: statistical error distributions and distance-dependent performance for seven methods (5–40 m).

Figure 7 further presents cumulative distribution functions (CDFs) and distance-dependent MAE curves for all seven methods. In the azimuth CDF (Figure 7a), MCS-AoA reaches the 90th percentile at roughly half the error of the next-best baseline, while the DNN-AoA curve displays a long tail indicative of poor worst-case performance. In the azimuth MAE versus distance plot (Figure 7c), all conventional methods (PDOA, MUSIC, MVDR, ESPRIT) see their MAE increase by 2–5× between 5 m and 40 m, whereas MCS-AoA rises by less than 50% over the same span. PwMUSIC demonstrates intermediate performance, consistent with the partial benefit afforded by amplitude weighting. In the elevation plots (Figure 7b,d), DNN-AoA incurs the highest overall elevation MAE (2.66°), reflecting limited generalization from the small training set (∼1500 samples). TLS-ESPRIT and MUSIC show comparable elevation accuracy, as both rely on subspace decomposition, but neither matches the credibility-weighted covariance of MCS-AoA, which keeps its elevation MAE below $2^{{}^{\circ}}$ at every tested distance.

### 5.3. Underground Logistics Tunnel (5–80 m)

**Figure 8:**
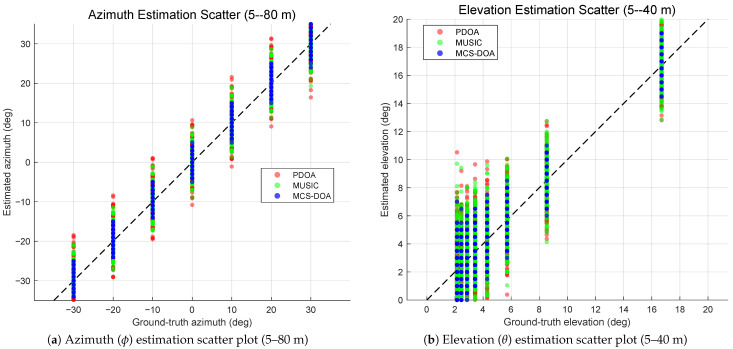
Underground logistics tunnel: AoA estimation scatter plots.

**Figure 9:**
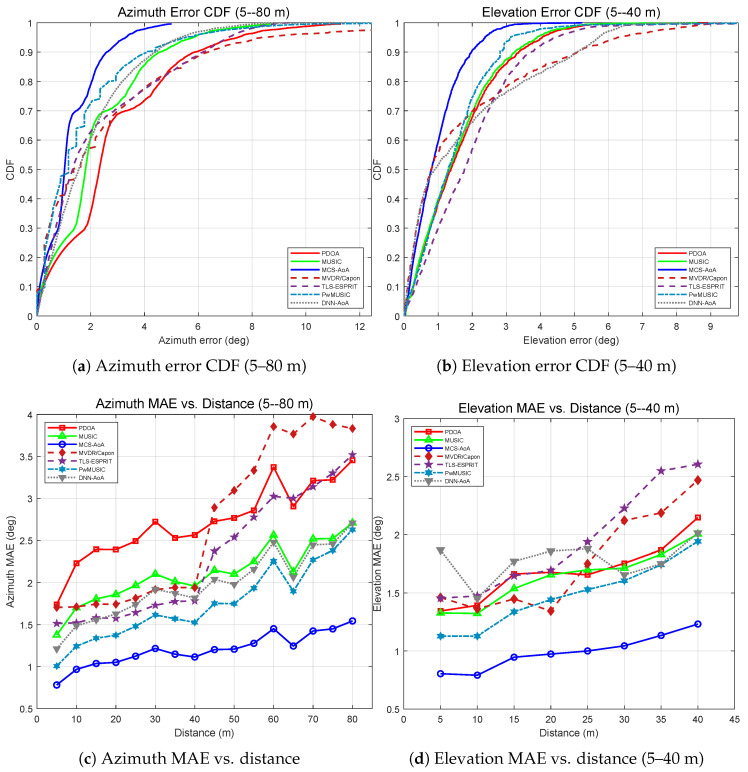
Underground logistics tunnel: statistical error distributions and distance-dependent performance for seven methods.

The method is further validated in an underground logistics tunnel. Figure 8a shows the azimuth estimation results over 5–80 m. MCS-AoA achieves an overall azimuth MAE of 1.19°—a 50% reduction relative to PDOA (2.40°) and a 36% reduction relative to MUSIC (1.87°). Table 10 reports the MAE for all seven methods. PwMUSIC (1.75°) outperforms MUSIC (1.87°), consistent with the corridor finding that path-weighted spectral estimation benefits from the strong direct path in confined environments. DNN-AoA (1.97°) follows, while TLS-ESPRIT (2.30°) and PDOA (2.40°) exhibit higher azimuth error. MVDR/Capon (2.70°) exhibits the highest azimuth MAE due to covariance ill-conditioning at long range. Elevation results over 5–40 m are presented in Figure 8b, where MCS-AoA attains an elevation MAE of 0.99° compared with PDOA (1.69°) and MUSIC (1.63°). PwMUSIC (1.48°) again ranks second, confirming its robustness in the elevation dimension. DNN-AoA (1.78°) and MVDR/Capon (1.77°) both exceed PDOA, while TLS-ESPRIT (1.95°) exhibits the highest elevation MAE owing to the single-source rank-one subspace assumption that becomes less accurate in the longer tunnel. Figure 9 summarizes the CDFs and distance-dependent MAE curves. Consistent with the corridor findings, MVDR/Capon shows significant degradation at longer range, and PwMUSIC consistently outperforms MUSIC while still falling short of MCS-AoA owing to its reliance on amplitude-only weighting.

Beyond the aggregate statistics, the measurements reveal clear distance-dependent behavior. Table 11 presents the range-segmented performance, and Table 12 and Table 13 provide per-distance breakdowns. Over 5–40 m, the azimuth MAE ranges from 0.78° to 1.22° and the elevation MAE from 0.79° to 1.23°; the 90th-percentile elevation error remains within $2^{{}^{\circ}}$. Over 45–80 m, the azimuth MAE ranges from 1.20° to 1.54°. Across the full 5–80 m span, the 90th-percentile azimuth error remains within 2.5°. These per-distance figures confirm that MCS-AoA keeps the azimuth MAE within 1.55° at every tested distance from 5 m to 80 m despite the severe multipath in this environment.

## 6. Discussion

Across both test environments, classical subspace variants—MVDR/Capon and ESPRIT—fail to improve upon conventional MUSIC, despite well-established theoretical advantages under idealized conditions. Dense, closely spaced multipath corrupts the sample covariance matrix, and neither method can distinguish reliable from unreliable path contributions. PwMUSIC introduces amplitude weighting and does narrow the azimuth gap (1.44° versus 1.76° for MUSIC), yet its elevation MAE (2.17°) is 49% higher than that of MCS-AoA (1.46°). In waveguide-like channels, reflected components routinely match or exceed the LOS in power, so amplitude alone cannot separate a reliable path from a misleading one. MCS-AoA avoids this pitfall by incorporating three additional consistency dimensions (TOF, phase–geometry, coherence) that are sensitive to geometric plausibility rather than signal strength alone.

The DNN-AoA results tell a complementary story. A 1.63° azimuth MAE in the corridor shows that data-driven approaches can learn useful CIR-to-angle mappings, yet the 2.66° elevation MAE—worst among all seven methods—exposes the generalization ceiling of a two-layer MLP trained on ∼1500 samples with leave-one-distance-out splits. Underground corridors present distance-dependent CIR variations that small datasets cannot cover adequately, a limitation that physics-guided credibility scoring avoids by design.

The three classical subspace/beamforming methods—MUSIC, TLS-ESPRIT, and MVDR/Capon—cluster tightly at 1.76°–1.79° azimuth MAE in the corridor (Table 9), an expected outcome when all three share the same sample covariance matrix and covariance estimation quality is the dominant bottleneck.

Compared with robust covariance techniques such as diagonal loading or shrinkage estimation, the credibility-weighted formulation (Equation (20)) operates at a different level: the former address ill-conditioning of the covariance matrix globally, whereas the latter discriminates among individual path contributions before constructing the covariance. The two strategies are complementary rather than competing.

Sparse reconstruction and compressive-sensing (CS)-based DOA methods offer an attractive alternative when the number of sources is small relative to a densely sampled spatial dictionary. However, their applicability to the present scenario is limited by two factors: (i) the physical array has only M=5 elements, which severely restricts the dictionary size and the achievable sparse-recovery performance; and (ii) the underground multipath environment produces a continuum of closely spaced arrivals rather than a few well-separated point sources, violating the sparsity assumption that underpins $\ell_{1}$-minimization and greedy pursuit algorithms. MCS-AoA circumvents these limitations by operating directly on per-tap CIR snapshots rather than requiring a spatial dictionary, and by leveraging physics-based credibility scoring to discriminate among candidate paths without relying on sparsity.

Simulation and field results agree qualitatively but differ in absolute magnitude. Three factors account for the gap: (i) channel non-stationarity and motion-induced CIR fluctuations absent from the parametric model; (ii) residual array calibration errors—sub-millimeter position deviations and 1–2° attitude misalignment—that perturb the phase–geometry consistency score; and (iii) hardware non-idealities including ADC quantization noise, clock jitter, and oscillator phase noise. Closing this gap motivates future work on online channel tracking, array self-calibration, and hardware-aware modeling. Despite this gap, MCS-AoA delivers the lowest MAE in both test environments (1.00°/1.46° in the corridor; 1.19° azimuth over 5–80 m in the logistics tunnel), and its 90th-percentile error is approximately half that of the next-best method.

The absolute accuracy levels can be contextualized against published UWB AoA benchmarks. Commercial dual-antenna systems based on the Qorvo DW3000 family typically achieve PDOA accuracies in the range of ±5–10° at short range (≤5 m) under indoor LOS conditions [29]; recent work by Martinelli et al. [30] reports an MAE of 5.21° at 3 m with a DW3220-based sensor node. Our PDOA baseline yields 2.46° (corridor) and 2.40° (tunnel), which is approximately 2× better and is physically attributable to the 10× increase in independent baselines (52=10 versus 1), coherent multi-channel sampling (<50 ps inter-channel jitter), and full 1016-tap CIR access. This comparison confirms that the baseline accuracy observed in our experiments is consistent with the hardware specifications rather than anomalous.

MCS-AoA is designed for the specific propagation regime found in narrow, elongated underground spaces—corridors, tunnels, utility galleries—where dense multipath with low excess delay and high relative amplitude is the dominant impairment. The approach should generalize to other confined settings with similar multipath profiles (e.g., indoor hallways, mine shafts), but in open environments where the LOS path is well separated from reflections, conventional subspace methods already perform adequately and the additional credibility machinery offers diminishing returns. The principal computational overhead is the 2D MUSIC spectrum search, which can be managed by limiting the candidate count *L* and employing a coarse-to-fine grid strategy.

## 7. Conclusions

Multipath structures in elongated underground environments need not degrade AoA accuracy—they can supply complementary spatial information. MCS-AoA exploits this by scoring every resolvable CIR component with a four-dimensional credibility metric and fusing the retained candidates through a weighted spatial covariance matrix, all without explicit LOS/NLOS classification. Field experiments confirm the practical impact: 1.00°/1.46° azimuth/elevation MAE in an underground corridor (5–40 m) and 1.19° azimuth MAE over 5–80 m in a logistics tunnel, outperforming all six baselines—PDOA, MUSIC, MVDR/Capon, TLS-ESPRIT, PwMUSIC, and DNN-AoA. Simulation results are consistent, with a 69.3% RMSE reduction over PDOA at 80 m. Design-choice validation further strengthens confidence: multiplicative credibility fusion outperforms additive and max-selection alternatives by 25–26%, and the algorithm is insensitive to its hyperparameters (peak threshold and risk weight vary performance by less than 0.1°). Taken together, the evidence supports physics-guided credibility weighting as a practical alternative to both classical subspace variants and data-driven methods in confined propagation environments where labeled training data are scarce.

## Figures and Tables

**Table 1:** Summary of credibility metric symbols.

Symbol	Name	Range	Physical Meaning
$S_{\mathrm{amp}}^{\left(\ell \right)}$	Amplitude significance	[0,1]	Relative peak power of candidate *ℓ* vs. strongest component
$S_{\mathrm{tof}}^{\left(\ell \right)}$	TOF proximity	(0,1]	Exponential penalty for excess delay relative to first arrival
$S_{\mathrm{geo}}^{\left(\ell \right)}$	Phase–geometry consistency	(0,1]	Agreement of inter-baseline phases with array geometry
$S_{\mathrm{coh}}^{\left(\ell \right)}$	Cross-baseline coherence	[0,1]	Mean resultant length of phase differences across baselines
$C_{\ell }$	Composite credibility	[0,1]	Product of four factors; used as fusion weight after thresholding

**Table 2:** MCS-AoA algorithm flow.

Step	Operation
Input	CIR matrix $R\in C^{N\times M}$, array geometry $\left\{p_{m}\right\}$, wavelength λ, and scan grids for ϕ and θ.
1	Candidate extraction (TOF gating): detect peaks on a reference channel with an amplitude threshold, keep at most L≤5 candidates in ascending delay order, and fall back to the global-maximum tap if no peak is detected.
2	Four-dimensional credibility evaluation for each candidate snapshot $x_{\ell }$: compute $S_{\mathrm{amp}}^{\left(\ell \right)}$, $S_{\mathrm{tof}}^{\left(\ell \right)}$, $S_{\mathrm{geo}}^{\left(\ell \right)}$, and $S_{\mathrm{coh}}^{\left(\ell \right)}$.
3	Credibility fusion: compute $C_{\ell }=S_{\mathrm{amp}}^{\left(\ell \right)}S_{\mathrm{tof}}^{\left(\ell \right)}S_{\mathrm{geo}}^{\left(\ell \right)}S_{\mathrm{coh}}^{\left(\ell \right)}$, select $\eta^{★}$, discard candidates with $C_{\ell }<\eta^{★}$, and normalize weights $w_{\ell }$ over the retained set.
4	Weighted covariance construction: $R_{w}=\sum_{\ell =1}^{L}w_{\ell }\;x_{\ell }x_{\ell }^{H}$.
5	Subspace decomposition: eigendecompose $R_{w}$ and form the noise subspace $E_{n}$.
6	MUSIC search: compute $P_{\mathrm{MUSIC}}\left(\phi ,\theta \right)$ over the scan grid and obtain $\left(\hat{\phi },\hat{\theta }\right)$ via peak search.
Output	Estimated azimuth/elevation $\left(\hat{\phi },\hat{\theta }\right)$.

**Table 3:** Key implementation parameters used in this work.

Parameter	Value	Role
Relative peak threshold	$0.15\;\max|r_{1}|$	Candidate extraction on the reference channel
Maximum candidate number *L*	5	Bounds the number of candidate snapshots
TOF decay factor α	0.1	Earliest-peak prior in $S_{\mathrm{tof}}^{\left(\ell \right)}$
$\lambda_{\mathrm{risk}}$	0.5	Risk weight in the adaptive threshold $\eta^{★}$
Scan grid for azimuth ϕ	−60°:1°:60°	2D MUSIC spectrum evaluation
Scan grid for elevation θ	−5°:0.5°:30°	2D MUSIC spectrum evaluation
Numerical stabilizer ϵ	$10^{-10}$	Prevents division by zero in normalization

**Table 4:** L-shaped antenna array parameters.

Parameter	Value	Description
Array elements	6 (1Tx + 5Rx)	L-shaped array
Receive channels for AoA	5	Used in AoA estimation
Minimum spacing $d_{\mathrm{short}}$	3.6 cm	Short-baseline sampling ($d_{\mathrm{short}}$ in Figure 1b)
Horizontal baseline $D_{1}$	22.5 cm	L-arm horizontal aperture ($D_{1}$ in Figure 1b)
Vertical baseline $D_{2}$	11.25 cm	L-arm vertical aperture ($D_{2}$ in Figure 1b)
Center frequency	3.9936 GHz	Center frequency

**Table 5:** Simulation accuracy comparison at representative distances (effective SNR ≈ 15 dB). The 40 m column reports the combined azimuth–elevation RMSE, while the 80 m column reports azimuth-only RMSE; the apparent improvement at 80 m reflects the single-angle evaluation rather than a distance-dependent accuracy gain.

Method	RMSE at 40 m (°)	RMSE at 80 m (°)
	(Azimuth + Elevation)	(Azimuth Only)
PDOA	3.20	2.31
MUSIC	1.82	1.60
MCS-AoA	0.91	0.71

**Table 6:** Ablation study: credibility fusion rule comparison (5–40 m simulation).

Fusion Rule	Azimuth MAE (°)	Elevation MAE (°)
Multiplicative (proposed)	1.10	0.60
Arithmetic mean (additive)	1.37	0.60
Maximum selection	1.39	0.58

**Table 7:** Hyperparameter sensitivity analysis: azimuth MAE (°) under individual parameter sweeps (5–40 m simulation).

Parameter	Swept Values				
α	0.01	0.05	0.10	0.20	0.50
Azimuth MAE	1.86	1.19	1.10	0.96	0.71
Threshold	0.05	0.10	0.15	0.20	0.25
Azimuth MAE	1.17	1.10	1.10	1.10	1.09
*L*	2	3	5	8	10
Azimuth MAE	0.90	1.03	1.10	1.10	1.10
$\lambda_{\mathrm{risk}}$	0.1	0.3	0.5	0.7	1.0
Azimuth MAE	1.11	1.11	1.10	1.11	1.13

**Table 8:** Extended baseline comparison (5–40 m simulation, median-per-point MAE).

Method	Azimuth MAE (°)	Elevation MAE (°)
PDOA	1.08	1.70
MUSIC	1.04	0.66
MVDR/Capon	1.46	0.76
TLS-ESPRIT	0.60	0.96
PwMUSIC	1.48	0.61
MCS-AoA (proposed)	1.10	0.60

**Table 9:** Measured AoA MAE in the underground corridor (5–40 m).

Method	Azimuth MAE (°)	Elevation MAE (°)
PDOA	2.26	2.49
MUSIC	1.76	2.40
MVDR/Capon	1.79	2.49
TLS-ESPRIT	1.76	2.34
PwMUSIC	1.44	2.17
DNN-AoA	1.63	2.66
MCS-AoA (proposed)	1.00	1.46

**Table 10:** Measured AoA MAE in the underground logistics tunnel (azimuth: 5–80 m; elevation: 5–40 m).

Method	Azimuth MAE (°)	Elevation MAE (°)
PDOA	2.40	1.69
MUSIC	1.87	1.63
MVDR/Capon	2.70	1.77
TLS-ESPRIT	2.30	1.95
PwMUSIC	1.75	1.48
DNN-AoA	1.97	1.78
MCS-AoA (proposed)	1.19	0.99

**Table 11:** Range-segmented AoA performance in the underground logistics tunnel.

Range/Estimation	Azimuth MAE (°)	Elevation MAE (°)
5–40 m (azimuth+elevation)	1.06	0.99
45–80 m (azimuth only)	1.34	–

**Table 12:** Per-distance near-range azimuth/elevation AoA performance in the underground logistics tunnel (5–40 m).

Distance (m)	Azimuth MAE (°)	Elevation MAE (°)
5	0.78	0.80
10	0.97	0.79
15	1.04	0.95
20	1.05	0.97
25	1.12	1.00
30	1.22	1.04
35	1.15	1.13
40	1.11	1.23
Average	1.06	0.99

**Table 13:** Per-distance mid-range azimuth-only AoA performance in the underground logistics tunnel (45–80 m).

Distance (m)	Azimuth MAE (°)
45	1.20
50	1.21
55	1.28
60	1.45
65	1.25
70	1.42
75	1.40
80	1.54
Average	1.34

## Data Availability

The raw CIR recordings (complex I/Q, 1016 taps per channel), ground-truth angle tables, and MATLAB (R2023a) processing scripts for all seven AoA estimation methods are available from the corresponding author upon reasonable request.

## Abbreviations

The following abbreviations are used in this manuscript:

UWB	Ultra-wideband
AoA	Angle of arrival
CIR	Channel impulse response
TOF	Time of flight
LOS	Line of sight
NLOS	Non-line of sight
PDOA	Phase difference of arrival
MUSIC	Multiple signal classification
MCS	Multipath credibility selection
MVDR	Minimum variance distortionless response
ESPRIT	Estimation of signal parameters via rotational invariance techniques
HRP	High-rate pulse

## References

[1] Zhou C., Jacksha R., Yan L., Reyes M., Kovalchik P. (2017). Time Domain and Frequency Domain Deterministic Channel Modeling for Tunnel/Mining Environments. Prog. Electromagn. Res. C.

[2] Nkakanou B., Delisle G.Y., Hakem N. (2011). Experimental Characterization of Ultra-Wideband Channel Parameter Measurements in an Underground Mine. J. Comput. Netw. Commun..

[3] Hrovat A., Kandus G., Javornik T. (2014). A Survey of Radio Propagation Modeling for Tunnels. IEEE Commun. Surv. Tutorials.

[4] Bashir S. (2014). Effect of Antenna Position and Polarization on UWB Propagation Channel in Underground Mines and Tunnels. IEEE Trans. Antennas Propag..

[5] Schmidt R. (1986). Multiple emitter location and signal parameter estimation. IEEE Trans. Antennas Propag..

[6] Roy R., Kailath T. (1989). ESPRIT—Estimation of Signal Parameters via Rotational Invariance Techniques. IEEE Trans. Acoust. Speech Signal Process..

[7] Capon J. (1969). High-Resolution Frequency-Wavenumber Spectrum Analysis. Proc. IEEE.

[8] Porozantzidou M.G., Chryssomallis M.T. (2010). Azimuth and elevation angles estimation using 2-D MUSIC algorithm with an L-shaped antenna. IEEE Antennas Wirel. Propag. Lett..

[9] Deng W., Li J., Tang Y., Zhang X. (2023). Low-Complexity Joint Angle of Arrival and Time of Arrival Estimation of Multipath Signal in UWB System. Sensors.

[10] Ledergerber A., D’Andrea R. (2019). Ultra-Wideband Angle of Arrival Estimation Based on Angle-Dependent Antenna Transfer Function. Sensors.

[11] Großwindhager B., Rath M., Kulmer J., Bakr M.S., Boano C.A., Witrisal K., Römer K. SALMA: UWB-based Single-Anchor Localization System using Multipath Assistance. Proceedings of the 16th ACM Conference on Embedded Networked Sensor Systems (SenSys ’18).

[12] Wielandt S., De Strycker L. (2017). Indoor Multipath Assisted Angle of Arrival Localization. Sensors.

[13] Nguyen H.A., Nguyen V.K., Witrisal K. (2022). Amplitude Modeling of Specular Multipath Components for Robust Indoor Localization. Sensors.

[14] Hu S., Guo L., Liu Z., Gao S. (2025). Multipath-Assisted Ultra-Wideband Vehicle Localization in Underground Parking Environment Using Ray-Tracing. Sensors.

[15] Marano S., Gifford W., Wymeersch H., Win M. (2010). NLOS Identification and Mitigation for Localization Based on UWB Experimental Data. IEEE J. Sel. Areas Commun..

[16] Zeng Z., Liu S., Wang L. NLOS Identification for UWB Based on Channel Impulse Response. Proceedings of the 2018 12th International Conference on Signal Processing and Communication Systems (ICSPCS).

[17] Stahlke M., Kram S., Mutschler C., Mahr T. NLOS Detection using UWB Channel Impulse Responses and Convolutional Neural Networks. Proceedings of the 2020 International Conference on Localization and GNSS (ICL-GNSS).

[18] Jiang C., Shen J., Chen S., Chen Y., Liu D., Bo Y. (2020). UWB NLOS/LOS Classification Using Deep Learning Method. IEEE Commun. Lett..

[19] Guvenc I., Chong C.C. (2009). A Survey on TOA Based Wireless Localization and NLOS Mitigation Techniques. IEEE Commun. Surv. Tutorials.

[20] Shen Y., Win M.Z. (2010). Fundamental Limits of Wideband Localization—Part I: A General Framework. IEEE Trans. Inf. Theory.

[21] Gezici S., Tian Z., Giannakis G.B., Kobayashi H., Molisch A.F., Poor H.V., Sahinoglu Z. (2005). Localization via Ultra-Wideband Radios: A Look at Positioning Aspects for Future Sensor Networks. IEEE Signal Process. Mag..

[22] Dardari D., Conti A., Ferner U., Giorgetti A., Win M.Z. (2009). Ranging With Ultrawide Bandwidth Signals in Multipath Environments. Proc. IEEE.

[23] Win M.Z., Scholtz R.A. (1998). Impulse Radio: How It Works. IEEE Commun. Lett..

[24] Molisch A.F. (2005). Ultrawideband Propagation Channels—Theory, Measurement, and Modeling. IEEE Trans. Veh. Technol..

[25] Dotlic I., Connell A., Ma H., Clancy J., McLaughlin M. Angle of Arrival Estimation Using Decawave DW1000 Integrated Circuits. Proceedings of the 2017 14th Workshop on Positioning, Navigation and Communications (WPNC).

[26] Smaoui N., Heydariaan M., Gnawali O. Single-Antenna AoA Estimation with UWB Radios. Proceedings of the 2021 IEEE Wireless Communications and Networking Conference (WCNC).

[27] Prince Mathew J., Nowzari C. (2024). ReLoki: A Light-Weight Relative Localization System Based on UWB Antenna Arrays. Sensors.

[28] Wymeersch H., Lien J., Win M.Z. (2009). Cooperative Localization in Wireless Networks. Proc. IEEE.

[29] Qorvo (2021). DW3000 User Manual: Calibration and Configuration; Application Note APS014.

[30] Martinelli A., Dolmans G., Luijten R., Stuijk S., Corporaal H. (2023). Angle of Arrival and Centimeter Distance Estimation on a Smart UWB Sensor Node. IEEE Trans. Instrum. Meas..

